# Comparison of Different Invasive Devices for the Treatment of Urinary Incontinence after Radical Prostatectomy

**DOI:** 10.1155/2022/8736249

**Published:** 2022-06-21

**Authors:** Stefano Salciccia, Pietro Viscuso, Giulio Bevilacqua, Antonio Tufano, Paolo Casale, Ettore De Berardinis, Giovanni Battista Di Pierro, Susanna Cattarino, Alessandro Gentilucci, Francesca Lourdes Lia, Di Giulio Ivan, Davide Rosati, Francesco Del Giudice, Alessandro Sciarra, Gianna Mariotti

**Affiliations:** ^**1**^ Department of Maternal-Infant and Urologic Sciences, “Sapienza” University of Rome, Policlinico Umberto I Hospital, Rome, Italy; ^**2**^ Department of Urology, Humanitas, Milan, Italy

## Abstract

**Purpose:**

To compare different forms of invasive treatments for postradical prostatectomy (RP) urinary incontinence (UI) in terms of quantitative and qualitative parameters and continence recovery rate.

**Methods:**

We distinguished five categories of treatment: *A* = bulking agents, *B* = fixed slings, *C* = adjustable slings, *D* = circumferential compressor devices (artificial sphincter), and *E* = noncircumferential compressor devices (ProACT). A literature search was performed following the PRISMA guidelines. We performed a cumulative meta-analysis to explore the trend in the effect sizes across groups at postoperative follow-up. We compared the available treatment arms using standardized mean difference (SMD) and event rate (ER) for questionnaire results, number of pads/day, and percentage of pad-free patients. *Evidence synthesis*. 36 clinical trials were selected. At baseline, in the different populations, mean number of pad-day varied from 1.1 to 8.8, 24-hour pad weight varied extremely from 17.3 *g* to 747.0 *g*, and mean ICIQ-UI-SF questionnaire score varied from 4.8 to 18.6. Considering a random effect model among eligible studies, ER of continence recovery was 0.33 (95% CI −0.12–0.78), 0.63 (95% CI 0.55–0.71), 0.65 (95% CI 0.58–0.72), 0.50 (95% CI 0.34–0.66), and 0.53 (95%CI 0.36–0.70), respectively, in groups A, B, C, D, and E (*I*^2^ 85.87%; *Q* 249.82—*P* > 0.01) (test of group differences *P*=0.22).

**Conclusion:**

In our analysis, the use of adjustable and fixed slings is associated with the highest whereas the use of bulking agents is associated with the lowest recovery rate of continence after treatment. Results are conditioned by an elevated rate of heterogeneity in part explained with a high variability of consistence in urinary leakage at baseline among populations.

## 1. Introduction

Prostate cancer is one of the most identified cancer types among men in the last decade, and the prevalence increases with age [1]. Despite the continuous improvement of surgical techniques and therapeutic offer, radical prostatectomy (RP) endures among the most related reasons of iatrogenic incontinence in men. While the postoperative urinary incontinence (UI) rates after prostatectomy for benign reason is 1%, a level of 5% to more than 40% has been reported after RP [2,3]. Sphincter deficiency and bladder dysfunction remain the recurring causes of UI after RP [2]. UI encountered after RP is mainly an early side effect that significantly impairs patient health quality of life. Behavioral therapeutic methods might always constitute the first step of treatment, and noninvasive therapies such as pelvic floor muscle exercises (PFME) or guided programs with biofeedback (BF) and/or functional electrical stimulation (ES) are usually attempted first [4–6]. Different invasive methods in men who failed these approaches or who are not considered for rehabilitative programs are recommended. Invasive surgical treatments are classified in five main groups [7]. Injection of bulking agents has been used to enhance the performance of harmed sphincter zone. EAU guidelines [7] underline that no differences among the different agents exist, and evidence that bulking agents can offer temporary and short-term improvement in UI is weak. Male slings can be divided into fixed devices positioned under the urethra through a retropubic or transobturator approach or adjustable slings where the tension can be adjusted postoperatively. In both cases, EAU guidelines [7] underline weak evidence to improve or to cure UI after RP and no evidence that adjustable versus fixed slings can offer additional benefits. External compressor devices can be distinguished in circumferential (artificial sphincter) and not circumferential (ProACT) compression of the urethral lumen. Artificial sphincter is considered effective to cure UI from the EAU guidelines [7] whereas limited short-term evidence is associated to ProACT [7]. The choice among these different invasive approaches can be obtained considering the degree of UI and urine leakage at baseline after RP. EAU guidelines suggest offering bulking agents only to men with mild incontinence who desire temporary relief, fixed, or adjustable slings in cases with mild-to-moderate leakage as well as compressor devices for moderate-to-severe UI [7]. Previous radiotherapy such as concomitant urethral strictures can significantly reduce the benefit from all these treatments, and a high risk of complications and need for explantation must be considered for compressor devices.

As stated by the international guidelines [7,8], data are still controversial, and the level of evidence remains uncertain. Therefore, we performed a systematic review and meta-analysis on the role of the different groups of invasive treatments in patients with post-RP UI.

## 2. Evidence Acquisition

### 2.1. Objective

Our objective is to analyze and compare different invasive treatments recommended in patients with post-RP UI. In particular, we distinguished five categories of treatment: *A* = bulking agents, *B* = fixed male slings, *C* = adjustable male slings, *D* = circumferential compression devices (artificial sphincter), and *E* = noncircumferential compressor devices (ProACT). We analyzed results in terms of UI improvement and continence recovery (pad-free status) after treatments using objective and subjective parameters available in clinical trials.

### 2.2. Search Strategy and Selection of the Studies

Our search in the literature of the last twenty years used electronic databases, such as PubMed, MEDLINE, Web of Science, Scopus, and the Cochrane Library. The process included the following items (“urinary incontinence” and “radical prostatectomy” and “bulking agents” and/or “male sling” and/or “compressor devices” and/or “artificial sphincter” and/or “ProACT”) according to the Preferred Reporting Items for Systematic review and Meta-Analyses (PRISMA) guidelines.

We considered original studies on clinical prospective trials analyzing patients submitted to RP with postsurgical UI. Two authors (PV and GB) independently evaluated titles and abstracts of all articles. The full-text articles were independently examined by three authors (AS, GB, and PV) so to define agreement with inclusion criteria. Following this process, two authors (GB and PV) extracted data from the selected articles. Final inclusion was evaluated by discussion of all investigators.

Inclusion criteria were as follows: (I) UI after RP; (II) at least one post-RP invasive treatment among bulking agents (A), fixed male sling (B), adjustable male sling (C), circumferential compressor device (D), and noncircumferential compressor device (E); (III) prospective analysis; and (IV) at least one of the following methods of evaluation: pad testing reporting pad weight or the number of daily pads and continence recovery rate (pad-free rate).

Exclusion criteria were as follows: (I) insufficient data for the outcomes reported as our objectives and (II) mixed populations without the possibility of data extraction.

### 2.3. Statistical Analysis

Risk of bias was assessed at the study level for each of the prospective cohort or randomized controlled studies included in full agreement with the Cochrane Collaboration's “Risk of bias” tool (Supplementary Table 1). We compared the available treatment arms using standardized mean difference (SMD) and event rate (ER) with 95% confidence interval (CI) at postoperative intervals following baseline evaluation. Heterogeneity of data was considered using the following[8]: (1) Cochran's Q-test with *P* < 0.05 signifying heterogeneity and (2) Higgins *I*^2^ test with inconsistency index.

A random effect model was used to calculate the pooled SMD and ER estimate for each group of treatment, and the results are presented as forest plots.

All analyses were performed through Stata version 1.7 (Stata Corporation, College Station, TX, USA) with all tests being two sided, and statistical significance was set at <0.05.

## 3. Evidence Synthesis

### 3.1. Studies Included in the Meta-Analysis

286 article references were initially considered. 162 were subsequently excluded due to duplication or not correspondence to the inclusion criteria. The remaining 125 articles were then reevaluated, and 89 did not meet the inclusion criteria. 36 remaining articles were included in our critical review and meta-analysis (Supplementary Figure 1, Table 1).

### 3.2. Quality of Studies and Sample Size

All 36 studies [9–43] were prospective mono- or multicenter clinical trials, and only 1 was randomized [9] (Table 1).

Size of populations with post-RP UI ranged from 4 to 173 cases. In all trials, the characteristics of the patient population were not accurately described in terms of either preoperative characteristics (preoperative lower urinary tract symptoms, prostate volume, PC stage, related diseases, or treatments), surgical techniques during RP, or postoperative noninvasive rehabilitative treatments that may influence UI. Stratification of results on the basis of these characteristics was not possible.

### 3.3. Assessment of Postoperative Complications

Outcomes were evaluated at variable postdevice follow-up intervals ranging from 1 to 12 months. Rates of intraoperative or postoperative severe complications requiring a new procedure or the removal of the device ranged from 0% to 5% in all four groups of devices. Only in two studies (19 and 42) higher rates of severe complications were found with 44.8% of 29 cases submitted to ProACT and 10.9% of 38 cases submitted to a fixed sling. Other nonsevere complications such as pain, infection, and acute urinary retention were common in all studies ranging from 0% to 95% of cases (Table 1).

### 3.4. Assessment of Continence Improvement

Post-RP continence status was mainly assessed using urinary symptom questionnaires, pad test results, and rate of pad-free cases. In particular, different questionnaires were used, and the ICIQ-UI-SF was the more completed in 14 studies (Table 2). Number of pads daily used and its variation after device placement were the main parameters reported among trials (24 trials) (Table 2). Fourteen studies reported results in terms of pad weight (in grams) using the 24-hour pad test (12 trials) or the 1-hour pad test (3 trials) (Table 2). In 30 studies, continence achievement was objectively defined as no-pad use (pad-free status) or <2 *g* at 24-hour pad test. Most of the trials performed a preoperative urodynamic assessment; however, this evaluation was mainly not considered to determine continence improvement (Table 2).

### 3.5. Baseline Characteristics of Populations

Mean age of populations ranged from 60.2 to 74.0 years. Baseline parameters before placement of the device for UI were reported at different intervals after radical prostatectomy, often not specified. In the 36 trials, a percentage ranging from 0% to 44% of cases was submitted to adjuvant radiotherapy (RT) before placement of the device for UI (Table 2).

At baseline, mean number of pad-day varied from 1.1 to 8.8, 24-hour pad weight varied extremely from 17.3 *g* to 747.0 *g*, and mean ICIQ-UI-SF questionnaire score varied from 4.8 to 18.6 (Table 3).

### 3.6. Categories of Invasive Treatments

In our analysis, invasive treatments were divided into 5 main categories: *A* = bulking agents, *B* = fixed male slings, *C* = adjustable male slings, *D* = circumferential compression devices, and *E* = noncircumferential compression devices. The different treatment arms included (A) bulking agents (porcine dermal collagen and polyacrylate polyalcohol copolymer) in only 2 trials, (B) fixed mal slings in 21, (C) adjustable male slings in 5, (D) artificial sphincter in 3, and (E) ProACT in 5 studies (Table 2). In each treatment, regimen different methods were used among studies, with different surgical techniques and materials. The heterogeneity in terms of devices was particularly evident in group B with different fixed male slings (Advance, Invance, Toms), less in the groups C with adjustable slings (ATOMS), D with artificial sphincter (AMS), and E with Pro ACT.

### 3.7. Outcome Results in terms of Number of Pads, Pad Weight, and ICIQ-UI-SF Questionnaire

Unfortunately, data regarding baseline and posttreatment values in terms of the number of pads, pad weight, and ICIQ-UI-SF questionnaires are often incomplete and heterogeneously presented as median with range or mean and SD. Among the five different groups of treatments, at baseline, mean values of the number of pads/day and questionnaire results were quite similar whereas 24-hour pad weight was strongly higher in studies on adjustable slings (Group C: mean baseline values ranging 681–747 *g*) when compared to the other groups (mean values = A: 17.3 *g*, B: 93 to 292 *g*, *D*: 135 *g*, and *E*: 345 to 543 *g*). The lack of postoperative data in terms of 24-hour pad weight does not consent to evaluate changes in this parameter after device placement. According to the number of pad/day, in all groups, a reduction after treatment was observed. Using the previously declared random effect model, we compared results among the 5 groups of invasive devices within eligible studies.

**Figure 1:**
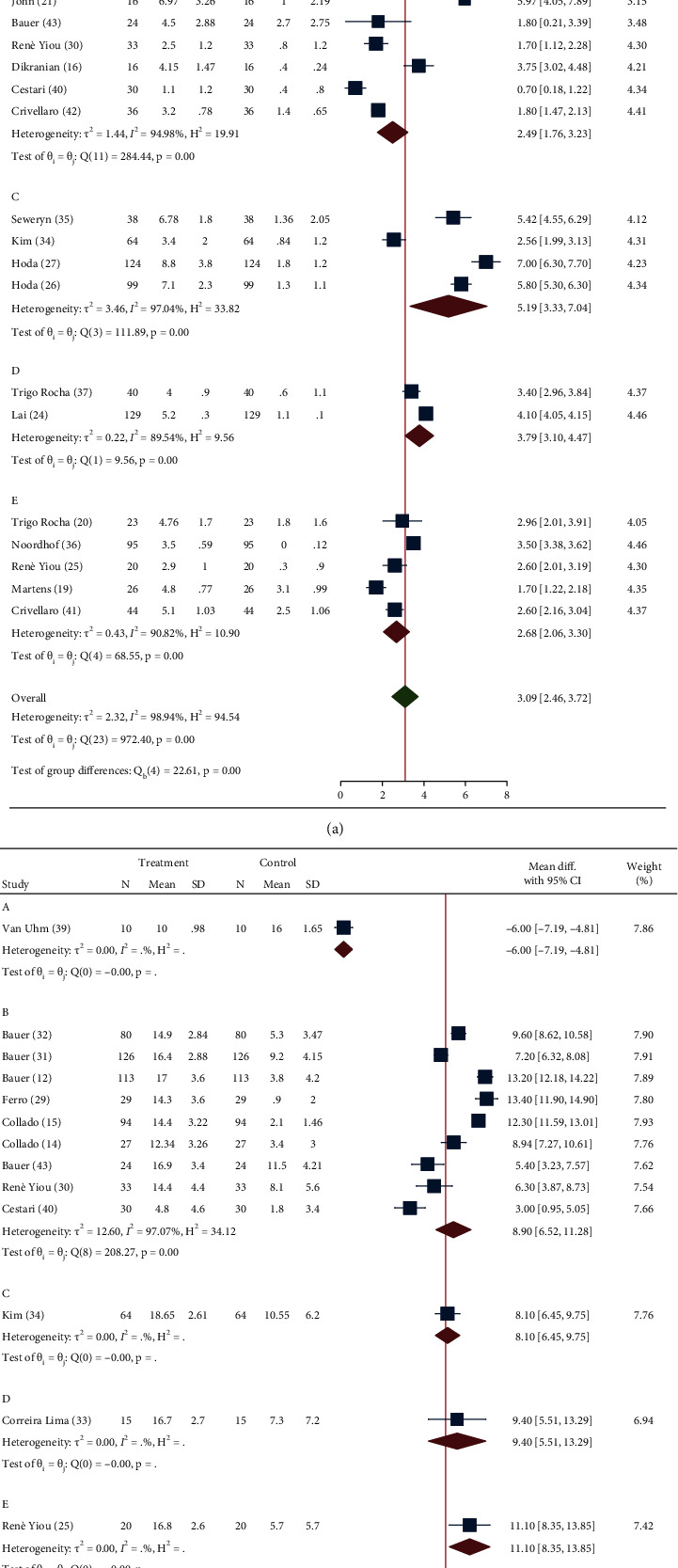
Forrest plot assessing standardized mean difference (SMD) for the number of pad/day (a) and ICIQ-SF score (b) recovery after device placement according to the five groups of invasive treatments for UI after RP, implemented within the studies included for analysis. (*A* = bulking agents, *B* = fixed slings, *C* = adjustable slings, *D* = circumferential compressor device, and *E* = noncircumferential compressor devices; SD = standard deviation; CI = confidence interval).

After placement of the device, pooled SMD for the reduction from baseline in the number of pad/day was significantly different with −2.0 (95% CI −1.43/−2.57), −2.49 (95% CI −1–76/−3.23), −5.19 (95% CI −3.33/−7.04), −3.79 (95% CI –3.10/−4.47), −2.68 (95% CI −2.06/−3.30), respectively, in groups A, B, C, D, and E (*I*^2^ 98.9%; *Q* 972.40—*P* < 0.01) (test of group differences *P* < 0.01) (Figure 1(a)). After placement of the device, pooled SMD for mean ICIQ-UI-SF score reduction from baseline was significantly different with 6.0 (95% CI 4.81/7.19), −8.90 (95% CI −6.52/−11.28), −8.10 (95% CI −6.45/−9.75), −9.40 (95% CI −5.51/−13.29), and −11.10 (95% CI −8.35/−13.85), respectively, in groups A, B, C, D, and E (*I*^2^ 98.32%; *Q* 857.18—*P* < 0.01) (test of group differences *P* < 0.01) (Figure 1(b)).

Deeks' funnel plots are displayed in Supplementary Figure 2, and meta-regression plots and analysis are presented in Supplementary Figure 3. We found an association between the baseline mean number of pad-day used and the subsequent improved SMD recovery after treatment as the possible cause for the consistent heterogeneity retrieved among the studies.

### 3.8. Outcome Results in terms of Continence Rate Recovery

**Figure 2:**
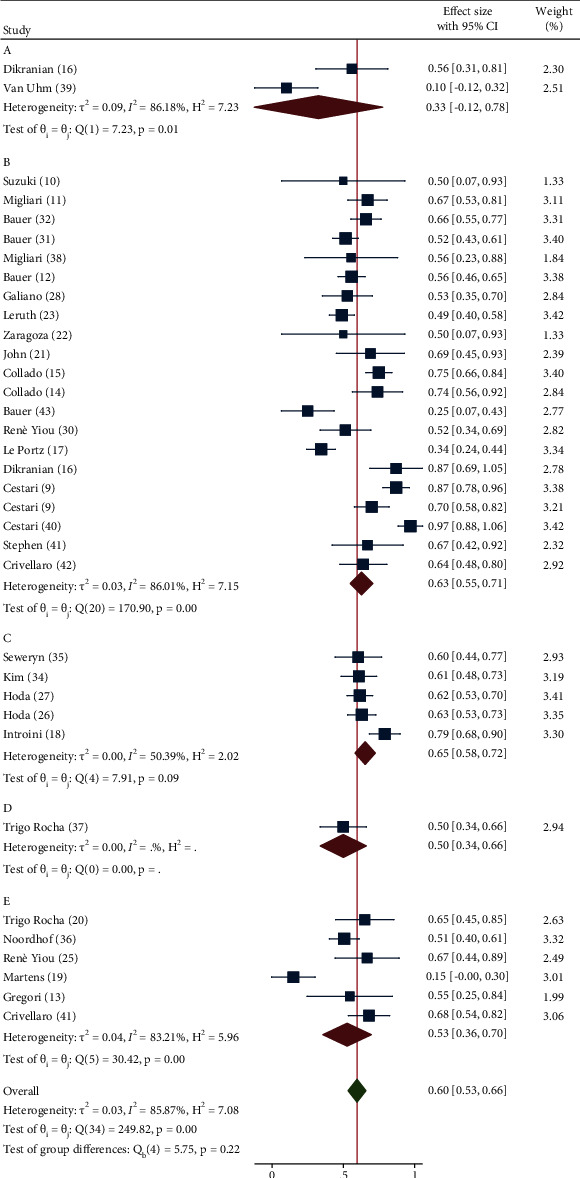
Forrest plot assessing pad-free event rate recovery after device placement according to the five groups of invasive treatments for UI after RP implemented within the studies included for analysis. (*A* = bulking agents, *B* = fixed slings, *C* = adjustable slings, *D* = circumferential compressor device, and *E* = noncircumferential compressor devices; SD = standard deviation; CI = confidence interval).

A meta-analysis was performed to examine the rate of a complete continence recovery (pad-free rate or pad weight <2 *g*) with 95% CI obtained after placement of the devices for UI among the five groups of treatment. According to a random effect model among eligible studies, ER of continence recovery was 0.33 (95% CI −0.12–0.78), 0.63 (95% CI 0.55–0.71), 0.65 (95% CI 0.58–0.72), 0.50 (95% CI 0.34–0.66), and 0.53 (95% CI 0.36–0.70), respectively, in groups A, B, C, D, and E (*I*^2^ 85.87%; *Q* 249.82—*P* > 0.01) (test of group differences *P*=0.22) (Figure 2).

Deeks' funnel plots are displayed in Supplementary Figure 2 and meta-regression plots and analysis are presented in Supplementary Figure 3. Again, we found an association between the baseline mean number of pad-day used and the ER of continence recovery after treatment as the possible cause for the consistent heterogeneity retrieved among the studies.

## 4. Discussion

This is the first meta-analysis on invasive treatments for post-RP UI, comparing results among five groups of devices as classified by the EAU guidelines [6]. A correct and standardized quantification of UI helps to define its impact on the quality of life of the patient and consents to assess treatment results. The evaluation of UI in patients should always combine objective quantitative and individual subjective parameters. International guidelines [6,7] do not precisely recommend how to monitor in clinical trials these two parameters: several questionnaires are mentioned, and quantification of leakage is associated to different diagnostic tools including pad tests. Either in trials on noninvasive rehabilitative treatments or in those on invasive therapies for UI after RP, validated questionnaires are always used, but data are extremely heterogeneous, and different questionnaires are used among studies.

Regarding the quantitative analysis of urine leakage, a different approach is considered comparing trials on noninvasive and invasive modalities. Almost all trials on rehabilitative techniques for UI include pad test (24-hour and in some cases 1- or 3-hour pad test) to quantify baseline, variations during follow-up, and definition of continence [3–5].

On the contrary, almost all trials on invasive techniques for UI after RP consider the daily determination of the number of pads as the primary toll to quantify leakage at baseline and to determine treatment efficacy [9–41]. There are no reasons to use different quantitative evaluations between noninvasive and invasive treatments for UI after RP, and this heterogeneity does not consent comparison. The determination of the number of pads is a less valid tool to quantify urine leakage and its variation during treatments, and it only consents to define a pad or no-pad status among patients. There exists an extreme variability in the use of pad among patients in relation of few drops or relevant leakages that negatively influence quantification of UI. Pad testing is a specific tool to quantify UI and to follow results during or after treatments for UI. A day (24-hour) pad test is a more reliable picture of a real-world situation for the patient, but it can be more influenced by variations in daily activities from different patients and different follow-up intervals. Urodynamic evaluation is limited in the use for invasive procedures, useful for the initial diagnosis but not for monitoring and quantifying leakage of urine after treatment.

As stated by international guidelines [6,7], data are still controversial, and the level of evidence remains uncertain. In the present meta-analysis, following the PRISMA statements, we selected 36 prospective studies on the use of invasive treatments for UI after RP corresponding to our inclusion criteria. After a first selection, 125 articles were evaluated; however, 89 of them were excluded mainly because they do not objectively reported results in terms of pad weight or number of daily pads or because mixed populations were included. The quality of data from these 36 trials was low with only one randomized study and sample sizes range from 4 to 173 cases. Several of these trials are mainly reported as a presentation of the surgical technique, and none of these studies accurately defined the patient population in terms of preoperative characteristics that may influence UI. Therefore, it was not possible to stratify our results based on pre-, intra-, or postoperative variables. Another relevant limitation is a heterogeneous and variable postdevice follow-up interval ranging from 1 to 12 months to evaluate improvement or resolution of UI. Moreover, in none of the 36 trials results were adequately stratified on the basis of previous radiotherapy (ranging from 0 to 44% of cases) or distinguishing in terms of mild, moderate, or severe UI.

Unexpectedly, in the populations considered in these trials, baseline urinary leakage strongly varied either in terms of mean number of pad-day (from 1.1 to 8.8), or 24-hour pad weight (from 17.3 *g* to 747.0 *g*), or mean ICIQ-UI-SF questionnaire score (from 4.8 to 18.6).

Our analysis found a significant heterogeneity of results either in terms of standard mean difference in number of pad and ICIQ questionnaire scores or in terms of event rate for recovery of continence after treatment (*I*^2^ > 80%). The consistence of urinary leakage at baseline is a variable able to condition the heterogeneity of results in terms of the different variables and in the different treatment groups.

The lack of considerable postoperative results in the selected studies in terms of pad weight does not consent to evaluate the effect of the different groups of devices on this parameter. More data are available in terms of number of pad/day, and in all groups, a significant (*P* < 0.01) mean reduction was observed after treatment, with the lowest improvement (−2.0 (95% CI −1.43/−2.57) pad/day) in the group of bulking agents and the highest (−5.19 (95% CI −3.33/−7.04) pad/day) in the group of adjustable slings, with a significant difference among groups (*P* < 0.01). Similarly, our meta-analysis shows a higher ER of continence recovery after fixed (0.63 (95% CI 0.55–0.71)) and adjustable slings (0.65 (95% CI 0.58–0.72)), intermediate after artificial sphincter (0.50 (95% CI 0.34–0.66)) and ProACT (0.53 (95% CI 0.36–0.70)), and considerably lower after bulking agents (0.33 (95% CI −0.12–0.78)), although differences did not reach statistical significance (*P*=0.22) (Table 4).

## 5. Conclusions

In our analysis on invasive treatments for UI following RP, the use of adjustable and fixed slings is associated with the highest whereas the use of bulking agents is associated with the lowest reduction in the number of pad/day and recovery rate of continence after treatment. However, results are conditioned by an elevated rate of heterogeneity in part explained with a high variability of consistence in urinary leakage at baseline among populations.

The quantitative evaluation of urinary leakage and its impact on the patient should be improved and better standardized in clinical trials. The daily number of pads should not be considered as primary end point whereas the quantitative analysis should be homogeneously obtained by pad test results.

Preoperative variables that may condition UI after RP, and results after device placement should be regularly addressed by studies so to consent an effective stratification of results.

## Figures and Tables

**Table 1:** 36 prospective clinical trials included in the analysis: main characteristics of the trials.

Author	Year	Study type	No. of patients	Treatment group (A, B, C, D, and E)	Device used	Total complication rate (%)	Severe complication rate (%)
Suzuki et al. [10]	2012	PT	4	B	Gynemesh bone anchored sling	62.0%	0%
Suzuki et al. [10]	2012	PT	4	B	Prolenemesh bone anchored sling	62.0%	0%
Migliari et al. [11]	2006	PT	49	B	Polypropylene sling	96.0%	0%
Bauer et al. [31]	2016	PT	115	B	Advance XP sling	6.0%	3.5%
Bauer et al. [30]	2010	PT	137	B	Advance sling	13, 9%	2.2%
Migliari et al. [37]	2003	PT	9	B	Polypropylene sling	55, 5%	0%
Bauer et al. [12]	2009	PT	124	B	Advance sling	12, 9%	0, 8%
Ferro et al. [28]	2016	PT	29	B	VIRTUE transobturator sling	58, 6%	0%
Galiano et al. [27]	2016	PT	52	B	TOMS transobturator sling	13, 5%	0%
Leruth et al. [23]	2012	PT	173	B	TOMS transobturator sling	25, 4%	0%
Zaragoza et al. [22]	2005	PT	4	B	INVANCE sling	0%	0%
John et al. [21]	2005	PT	16	B	Porcine skin collagen + polypropylene sling	25.0%	0%
Collado et al. [15]	2018	PT	94	B	Advance + advance XP sling	23.4%	0%
Collado et al. [14]	2009	PT	27	B	Invance sling	26.0%	0%
Trigo Rocha et al. [36]	2008	PT	40	D	AMS 800 artificial sphincter	10.0%	0%
Correia Lima et al. [32]	2018	PT	15	D	BR—SL—AS—904 artificial sphincter	0%	0%
Lai et al. [24]	2009	PT	129	D	AMS 800 artificial sphincter	—	—
Trigo Rocha et al. [20]	2006	PT	25	E	ProACT	17.3%	0%
Bauer et al. [41]	2011	PT	24	B	Advance sling	16.7%	0%
Noordhof et al. [35]	2017	PT	143	E	PRO-ACT	9.8%	2.1%
Seweryn et al. [34]	2012	PT	38	C	ATOMS readjustable transobturator sling	52, 6%	0%
Kim et al. [33]	2016	PT	64	C	MRS readjustable sling	9, 4%	4, 7%
Renè Yiou et al. [25]	2014	PT	20	E	PRO-ACT	10.0%	0%
Renè Yiou et al. [29]	2016	PT	40	B	TOMS transobturator sling	17, 5%	0%
Hoda et al. [26]	2012	PT	124	C	ATMOS readjustable transobturator sling	60, 5%	4.0%
Hoda et al. [26]	2012	PT	99	C	ATMOS readjustable transobturator sling	68, 7%	4.0%
Martens et al. [19]	2009	PT	29	E	PRO-ACT	68.0%	44, 8%
Introini et al. [18]	2012	PT	66	C	Silimed periurethral constrictor adjustable sling	4, 5%	0%
Le Portz et al. [17]	2016	PT	93	B	Surgimesh M-SLING	2, 1%	0%
Dikranian et al. [16]	2004	PT	20	A	Porcine dermal Collagene	5.0%	5.0%
Dikranian et al. [16]	2004	PT	16	B	Silicone mesh sling	12.0%	0%
Gregori et al. [13]	2008	PT	11	E	ProAct	0%	0%
Cestari et al. [9]	2017	RT	120	B	Autologous sling (6 branches versus 2 branches)	6.7%	5.0%
Van Uhm et al. [38]	2018	PT	10	A	Opsys bulking agent (polyacrylate polyalcohol copolymer	40.0%	0%
Cestari et al. [39]	2015	PT	60	B	Autologous sling	10.0%	0%
Stephen J et al. [40]	2005	PT	15	B	Sling polyglactin mesh	0%	0%
Crivellaro et al. [21]	2008	PT	46	E	Pro-ACT	12.3%	4%
Crivellaro et al. [21]	2008	PT	38	B	BAMS polypropylene bulbourethral sling	19.4%	10.9%
Queissert F et al. [43]	2022	PT	12	C	ATMOS readjustable transobturator sling	—	—

*Note*. PT = prospective nonrandomized trial. RT = randomized trial. Treatment group: *A* = bulking agent, *B* = fixed sling, *C* = adjustable sling, *D* = artificial sphincter, *E* = ProACT.

**Table 2:** 36 prospective clinical trials included in the analysis: main characteristics of the population. Number of cases; mean ± SD or median and (range).

Author	No. of patients	Treatment groups (A, B, C, D, and E)	Mean age (years)	BMI	Follow-up (months)	Adjuvant RT (% pz)	Measurements
Suzuki et al. [10]	4	B	70.6 (64–76)	—	6	ND	n pad/die, urodynamic
Suzuki et al. [10]	4	B	70.6 (64–76)	—	6	ND	n pad/die, urodynamic
Migliari et al. [11]	49	B	70.5 (65–75)	—	32	ND	n pad/die, urodynamic
Bauer et al. [31]	115	B	69.0 (47–82)	—	36	ND	24 h Pad test; ICIQ-UI; urodynamic
Bauer et al. [30]	137	B	69.5 (56–82)	—	27	13.5	1 h Pad test; n pad/die; urodynamic;
Migliari et al. [37]	9	B	74.0 (66–80)	—	6	ND	% Pad free, urodynamic
Bauer et al. [12]	124	B	68.9 (54–87)	—	6	13.0	n pad/1-hour pad test. 24-hour pad test
Ferro et al. [28]	29	B	65.5 ± 4.7	24.7	36	0	n pad, 24 h pad test, ICQ-SF, urodynamic
Galiano et al. [27]	52	B	64.9 ± 5.1	27.6 ± 3.6	12	8.8	n pad/die
Leruth et al. [23]	173	B	67.7 ± 7.3 (46–83)	26.6 ± 4.9	60	12.1	% Pad free, urodynamic
Zaragoza et al. [22]	4	B	65.0 (58–72)	—	12	ND	n pad/die
John et al. [21]	16	B	67.0 (56–83)	—	14	ND	n pad/die, urodynamic
Collado et al. [15]	94	B	66.0 (52–80)	27.5 (21–39)	49	ND	n pad/die 24 h pad test
Collado et al. [14]	27	B	66.0 (48–72)	—	18	ND	n pad/die, ICIQ-UI-SF, urodynamics
Trigo Rocha et al. [36]	40	D	68.3 ± 6.3	—	53.4	0	VAS score; n pad/die; urodynamic
Correia Lima et al. [32]	15	D	68.2 ± 7.5	26.61 ± 4.1	19.7	ND	Pad weight test, ICIQ - SF,
Lai et al. [24]	129	D	69.0 ± 0.6	—	34.1	26.0	n pad/die, urodynamic
Trigo Rocha et al. [20]	25	E	68.6	—	22.4	ND	n pad/die, urodynamic
Bauer et al. [41]	24	B	71.0 (61–77)	—	18	100	n pad/die, 1 h pad weigh, ICIQ-UI-SF
Noordhof et al. [35]	143	E	69.0 (66–73)	26,1 (24.1–28.1)	46	ND	n pad/die
Seweryn et al. [34]	38	C	70.0 (60–83)	—	17	44.7	n pad/die, urodynamic
Kim et al. [33]	64	C	69.58 ± 7.52	—	46	12.0	n pad/die
Renè Yiou et al. [25]	20	E	68.6 ± 9.0	—	12	ND	n pad, ICIQ-SF
Renè Yiou et al. [29]	40	B	67.7 ± 7.0	—	24	5.0	UCLA-PCI; ICIQ-SF;; n pad/die
Hoda et al. [26]	124	C	71.2 ± 5.5	—	19.1	35.0	n pad, urodynamic
Hoda et al. [26]	99	C	70.4 (55–86)	—	30	31.0	24 h Pad test, n pad/die, urodynamic
Martens et al. [19]	29	E	65.0 (61–75)	—	41	ND	n pad/die
Introini et al. [18]	66	C	66.0 (52–79)	—	26	7.5	% Pad free
Le Portz et al. [17]	93	B	72.5 ± 6.5	26.2	24	ND	n pad/die, 24 h pad test, urodynamic
Dikranian et al. [16]	20	A	64.8 (56–78)	—	6	5.5	Questionnaire, n pad/die, urodynamic
Dikranian et al. [16]	16	B	62.8 (63–72)	—	6	2.7	Questionnaire, n pad/die, urodynamic
Gregori et al. [13]	11	E	69.9 (64–77)	—	8	9.1	24 h Pad test
Cestari et al. [9]	120	B	64.0 (51–79)	25.6 (21.1–31.2)	12	0	n pad/die, ICIQ-UI-SF
Van Uhm et al. [38]	10	A	67.0 ± 6.1	29.7 ± 6.3	6	0	24 h Pad weight, ICIQ-SF, urodynamics
Cestari et al. [39]	60	B	65.0 (60–72)	25.3	12	ND	n pad/die, ICIQ-UI-SF
Stephen J et al. [40]	15	B	60.2 (49–71)	—	12	ND	n pad/die
Crivellaro et al. [21]	46	E	67.0 (45–82)	—	19	ND	n pad/die, UCLA, urodynamic
Crivellaro et al. [21]	38	B	65.0 (30–81)	—	33	ND	n Pad/die, UCL, urodynamic
Queissert et al. [43]	12	C	69.0 (64–72)	26.4	12	ND	Pad weight, n pad/die, Urodynamic, ICIQ-UI-SF

*Note*. ND = not defined. Treatment group: *A* = bulking agent, *B* = fixed sling, *C* = adjustable sling, *D* = artificial sphincter, and *E* = ProACT. Grey horizontal rows represent the second arm of treatment in the same trial.

**Table 3:** 36 prospective clinical trials included in the analysis: baseline values. Number of cases; mean ± SD or median and (range).

Author	No. of patients	Treatment groups (A, B, C, D, and E)	N pad/day (pre)	1 h pad test (g) (pre)	24 h pad test (g) (pre)	% Severe UI (>6) (pre)	% Moderate UI (3–5) (pre)	% Mild UI (1–2) (pre)	ICIQ-UI-SF (pre)
Suzuki et al. [10]	4	B	4.0 ± 0.8	—	—	—	—	—	—
Suzuki et al. [10]	4	B	3.5 ± 1.3	—	—	—	—	—	—
Migliari et al. [11]	49	B	—	—	—	25.0%	69.0%	6.0%	—
Bauer et al. [31]	115	B	—	—	272.0 (42–1600)	—	—	—	14.9 (8–22)
Bauer et al. [30]	137	B	4.9 (1–24)	124.4 (11–585)	—	31.0%	52.3%	16.7%	16.4 (5–22)
Migliari et al. [37]	9	B	—	—	—	—	—	—	—
Bauer et al. [12]	124	B	4.0 ± 1.1	119.5	292.2 (45–1200)	24.2%	48.4%	15.3%	17.0 ± 3.6
Ferro et al. [28]	29	B	2.2 ± 1.4	—	128.6 ± 71.9	—	72.4%	27.6%	14.3 ± 3.6
Galiano et al. [27]	52	B	2.2 ± 1.0	—	123.5 ± 107.8	—	—	—	—
Leruth et al. [23]	173	B	—	—	—	48,6%	30.0%	21.4%	—
Zaragoza et al. [22]	4	B	4.0 (3–5)	—	—	—	100%	—	—
John et al. [21]	16	B	7.0 (2–12)	—	—	38.0%	—	—	—
Collado et al. [15]	94	B	—	—	93.0 (12–507)	—	—	—	14.4 (5–21)
Collado et al. [14]	27	B	1.9 (1–3)	—	—	—	—	—	12.3 (8–21)
Trigo Rocha et al. [36]	40	D	4.0 ± 0.9 (3–10)	—	—	87,5%	12.5%	—	—
Correia Lima et al. [32]	15	D	—	—	135.19 ± 159.54	—	—	—	16.7 ± 2.7
Lai et al. [24]	129	D	5.2 ± 0.3 (1–15)	—	—	—	—	—	—
Trigo Rocha et al. [20]	25	E	4.7 ± 1.7	—	—	—	—	—	—
Bauer et al. [41]	24	B	4.5 (1.5–12)	89.5 (21–150)	—	—	—	—	16.9 (5–22)
Noordhof et al. [35]	143	E	3.5 (2–5)	—	—	35.0%	39.8%	25,.%	—
Seweryn et al. [34]	38	C	6.7 (2–10)	—	747.0 (230–1600)	57.8%	34.2%	7.9%	—
Kim et al. [33]	64	C	3.4 ± 2.0	—	—	14.1%	43.8%	42.2%	18.6 ± 2.61
Renè Yiou et al. [25]	20	E	2.9 ± 1.0	—	345.1 ± 308.4	—	—	—	16.8 ± 2.6
Renè Yiou et al. [29]	40	B	2.5 ± 1.2	—	—	—	—	—	14.4 ± 4.4
Hoda et al. [26]	124	C	8.8 ± 3.8 (3–18)	—	725 ± 372 (110–2300)	69.6%	30.4%	0%	—
Hoda et al. [26]	99	C	7.1 (3–12)	—	681.0 (100–2000)	70.7%	29.3%	0	
Martens et al. [19]	29	E	4.8 (3–6)	—	—	—	—	—	—
Introini et al. [18]	66	C	—	—	—	—	—	—	—
Le Portz et al. [17]	93	B	1.8 (1–4)	—	109.1 ± 116.37	—	—	—	—
Dikranian et al. [16]	20	A	3.4 (2–6)	—	—	—	—	—	—
Dikranian et al. [16]	16	B	4.0 (2–7)	—	—	—	—	—	—
Gregori et al. [13]	11	E	—	—	543.6 (80–1300)	18.0%	64.0%	18.0%	—
Cestari et al. [9]	120	B	—	—	—		—	—	—
Van Uhm et al. [38]	10	A	—	—	17.3 (6.4–20.9)	—	—	—	10.0 (9.0–12.0)
Cestari et al. [39]	60	B	1.1 ± 1.2	—	—	—	—	—	4.8 ± 4.6
Stephen J et al. [40]	15	B	—	—	—	—	—	—	—
Crivellaro et al. [21]	46	E	5.1 (5–2)	—	—	—	89.0%	11.0%	—
Crivellaro et al. [21]	38	B	3.2 (3–1)	—	—	—	72.0%	28.0%	—
Queissert et al. [43]	12	C	4.0	—	240 (72–125)	—	—	—	16.0

*Note*. UI = urinary incontinence. Treatment group: *A* = bulking agent, *B* = fixed sling, *C* = adjustable sling, *D* = artificial sphincter, and *E* = ProACT. Grey horizontal rows represent the second arm of treatment in the same trial. Severe UI is defined as > 6 UI episodes, moderate 3–5 UI episodes, and mild 1–2 UI episodes daily.

**Table 4:** 36 prospective clinical trials included in the analysis: posttreatment results. Number of cases; mean ± SD or median and (range).

Author	N of patients	Treatment groups (A, B, C, D, and E)	N pad/day (post)	1 h pad test (g) (post)	24 h pad test (g) (post)	ICIQ-UI-SF (post)	% Pad free (post)
Suzuki et al. [10]	4	B	1.8 ± 1.3	—	—	—	50%
Suzuki et al. [10]	4	B	0.5 ± 0.6	—	—	—	50%
Migliari et al. [11]	49	B	—	—	—	—	67%
Bauer et al. [31]	115	B	—	—	24.7 (0–258)	5.3 (0–17)	68.8%
Bauer et al. [30]	137	B	2.1 (0–20)	47.6 (0–320)	—	9.2 (0–21)	51.6%
Migliari et al. [37]	9	B	—	—	—	—	55.5%
Bauer et al. [12]	124	B	0 ± 0.5	8.6 (0–45)	13.4 (0–125)	3.8 ± 4.2	55.8%
Ferro et al. [28]	29	B	0.3 ± 0.5	—	2.6 ± 5.4	0.9 ± 2.0	
Galiano et al. [27]	52	B	0.7 ± 0.9	—	43.4 ± 109.9	—	52.9%
Leruth et al. [23]	173	B	—	—	—	—	49%
Zaragoza et al. [22]	4	B	—	—	—	—	50%
John et al. [21]	16	B	1.0 (0–10)	—	—	—	69%
Collado et al. [15]	94	B	—	—	—	2.1 (0–7)	75%
Collado et al. [14]	27	B	—	—	29.6 (19–40)	3.4 (0–13)	
Trigo Rocha et al. [36]	40	D	0.6 ± 1.1	—	—	—	50%
Correia Lima et al. [32]	15	D	—-	—	75.72 ± 95.29	7.3 ± 7.2	
Lai et al. [24]	129	D	1.1 ± 0.1 (0–8)	—	—	—	
Trigo Rocha et al. [20]	25	E	1.8 ± 1.6	—	—	—	65%
Bauer et al. [41]	24	B	2.7 (0–12)	47 (0–138)	—	11.5 (0–21)	25%
Noordhof et al. [35]	143	E	0.2 (0–2)	—	—	—	50.6%
Seweryn et al. [34]	38	C	1.4 (0–10)	—	115.0 (0.1500)	—	60.5%
Kim et al. [33]	64	C	0.8 ± 1.2	—	—	10.55 ± 6.2	60.9%
Renè Yiou et al. [25]	20	E	0.3 ± 0.9	—	—	5.7 ± 5.7	66.7%
Renè Yiou et al. [29]	40	B	0.8 ± 1.2	—	—	8.1 ± 5.6	51.5%
Hoda et al. [26]	124	C	1.8 ± 1.2 (0–7)	—	—	—	61.6%
Hoda et al. [26]	99	C	1.3 (0–8)	—	79.7 (0–285)	—	63%%
Martens et al. [19]	29	E	3.1 (0–5)	—	—	—	31%
Introini et al. [18]	66	C	—	—	—	—	79%
Le Portz et al. [17]	93	B	—	—	40 (0–185)	—	34.4%
Dikranian et al. [16]	20	A	1.4 (0–2)	—	—	—	56%
Dikranian et al. [16]	16	B	0.4 (0–1)	—	—	—	87%
Gregori et al. [13]	11	E	—	—	17 ± 2.7	—	
Cestari et al. [9]	120	B	—	—	—	1.8±-3.1	98%
Van Uhm et al. [38]	10	A	—	—	40.3 (5.9–130.6)	16.0 (12.5–17.5)	10%
Cestari et al. [39]	60	B	0.4 ± 0,8	—	—	1.8 ± 3.4	97%
Stephen J et al. [40]	15	B	—	—	—	—	67%
Crivellaro et al. [21]	46	E	2.5 (0–5)	—	—	—	68%
Crivellaro et al. [21]	38	B	1.4 (0–3)	—	—	—	64%
Queissert et al. [43]	12	C	0.9	—	70.0 (0–700.0)	5.5	75%

*Note*. Treatment group: *A* = bulking agent, *B* = fixed sling, *C* = adjustable sling, *D* = artificial sphincter, and *E* = ProACT. Grey horizontal rows represent the second arm of treatment in the same trial.

## Data Availability

Meta-analysis data are enclosed as supplementary files.

## Abbreviations

RP: Radical prostatectomy
PC: Prostate cancer
UI: Urinary incontinence
ICIQ-UI-SF: International Consultation on Incontinence Questionnaire, Urinary Incontinence short form
EAU: European Association of Urology
PRISMA: Preferred Reporting Items for Systematic Review and Meta-Analyses
SMD: Standardized mean difference
ER: Event rate
CI: Confidence interval

## Supplementary Materials

Supplementary Materials Supplementary Figure 1. Flow chart for meta-analysis (PRISMA). Supplementary Figure 2. Deeks' funnel plots for standardized mean difference (SMD) of number of pad/day (a), ICIQ-SF score (b), and for continence event rate recovery (c) at follow-up. Supplementary Figure 3. Meta-regression plots in relation to standardized mean difference (SMD) for the number of pad (a), ICIQ-SF score (b), and for continence event rate (c) recovery at follow-up. Supplementary Table 1. Risk of Bias for all studies included in the meta-analysis. PRISMA checklist: checklist reporting location in the manuscript of the different items related to PRISMA analysis.
