# Supplementary material for: Comparison of Different Invasive Devices for the Treatment of Urinary Incontinence after Radical Prostatectomy
Source: Adv Urol. 2022 Jun 21;2022:8736249. doi: 10.1155/2022/8736249 (PMC9239822; doi:10.1155/2022/8736249)
Supplement: Supplementary Materials — Supplementary Figure 1. Flow chart for meta-analysis (PRISMA). Supplementary Figure 2. Deeks' funnel plots for standardized mean difference (SMD) of number of pad/day (a), ICIQ-SF score (b), and for continence event rate recovery (c) at follow-up. Supplementary Figure 3. Meta-regression plots in relation to standardized mean difference (SMD) for the number of pad (a), ICIQ-SF score (b), and for continence event rate (c) recovery at follow-up. Supplementary Table 1. Risk of Bias for all studies included in the meta-analysis. PRISMA checklist: checklist reporting location in the manuscript of the different items related to PRISMA analysis. [file 8736249.f1.zip › 8736249.f1/Supplementary fig 1 final (1).pdf]

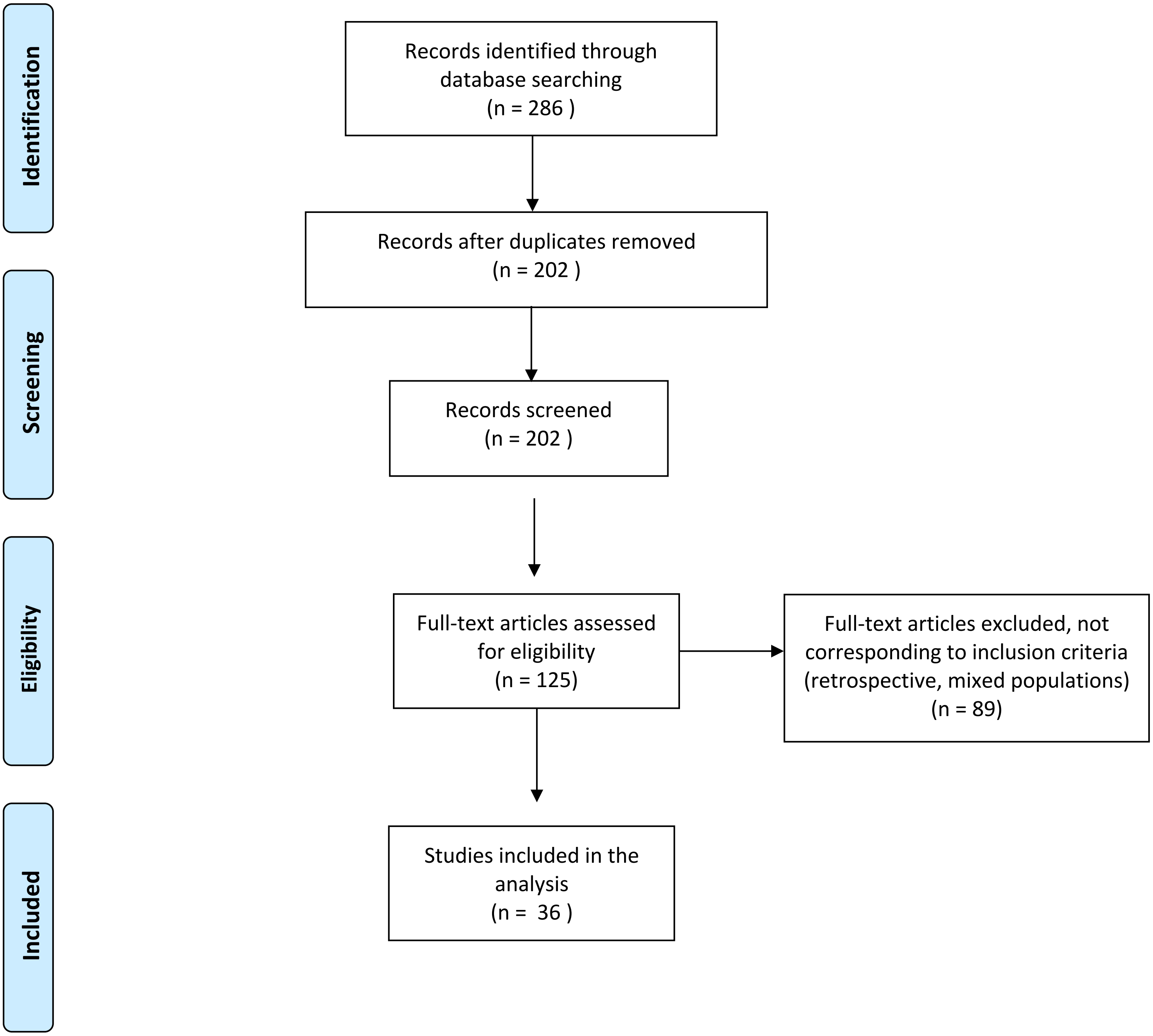

From: Moher D, Liberati A, Tetzlaff J, Altman DG, The PRISMA Group (2009). *P*referred *R*eporting *I*tems for *S*ystematic Reviews and *M*eta-*A*nalyses: The PRISMA Statement. PLoS Med 6(7): e1000097. doi:10.1371/journal.pmed1000097

For more information, visit www.prisma-statement.org.
