# Supplementary material for: Comparison of Different Invasive Devices for the Treatment of Urinary Incontinence after Radical Prostatectomy
Source: Adv Urol. 2022 Jun 21;2022:8736249. doi: 10.1155/2022/8736249 (PMC9239822; doi:10.1155/2022/8736249)
Supplement: Supplementary Materials — Supplementary Figure 1. Flow chart for meta-analysis (PRISMA). Supplementary Figure 2. Deeks' funnel plots for standardized mean difference (SMD) of number of pad/day (a), ICIQ-SF score (b), and for continence event rate recovery (c) at follow-up. Supplementary Figure 3. Meta-regression plots in relation to standardized mean difference (SMD) for the number of pad (a), ICIQ-SF score (b), and for continence event rate (c) recovery at follow-up. Supplementary Table 1. Risk of Bias for all studies included in the meta-analysis. PRISMA checklist: checklist reporting location in the manuscript of the different items related to PRISMA analysis. [file 8736249.f1.zip › 8736249.f1/Supplementary Table 1 (2).docx]

**Supplementary Table 1**. Risk of Bias for all studies included in the meta-analysis.

| **RISK OF BIAS** | | | | |
| --- | --- | --- | --- | --- |
|  | **Patient selection** | **index tests** | **Reference standard** | **Flow and timing** |
| **Suzuki Et Al (10)** | LOW | UNCLEAR | LOW | LOW |
| **Migliari Et Al (11)** | UNCLEAR | LOW | UNCLEAR | LOW |
| **Bauer Et Al (31)** | LOW | LOW | UNLCEAR | LOW |
| **Bauer Et Al (30)** | LOW | HIGH | LOW | UNCLEAR |
| **Migliari Et Al (37)** | LOW | UNCLEAR | LOW | LOW |
| **Bauer Et Al (12)** | LOW | UNCLEAR | UNCLEAR | LOW |
| **Ferro Et Al (28)** | LOW | UNCLEAR | LOW | UNCLEAR |
| **Galiano Et Al (27)** | HIGH | UNCLEAR | LOW | LOW |
| **Leruth Et Al (23)** | LOW | UNCLEAR | LOW | HIGH |
| **Zaragoza Et Al (22)** | UNCLEAR | UNCLEAR | LOW | LOW |
| **John Et Al (21)** | UNCLEAR | LOW | UNCLEAR | LOW |
| **Collado Et Al (15)** | UNCLEAR | LOW | UNCLEAR | LOW |
| **Collado Et Al (14)** | UNCLEAR | LOW | LOW | UNCLEAR |
| **Trigo Rocha Et Al (36)** | LOW | UNCLEAR | LOW | UNCLEAR |
| **Correia Lima Et Al (32)** | HIGH | LOW | UNCLEAR | LOW |
| **Lai Et Al (24)** | LOW | LOW | UNCLEAR | LOW |
| **Trigo Rocha Et Al (20)** | LOW | UNCLEAR | LOW | LOW |
| **Bauer Et Al (42)** | LOW | UNCLEAR | LOW | UNCLEAR |
| **Noordhof Et Al (35)** | UNCLEAR | LOW | UNCLEAR | LOW |
| **Seweryn Et Al (34)** | HIGH | LOW | LOW | LOW |
| **Kim Et Al (33)** | LOW | HIGH | LOW | LOW |
| **Renè Yiou Et Al (25)** | LOW | LOW | UNCLEAR | UNCLEAR |
| **Renè Yiou Et Al (29)** | UNCLEAR | LOW | UNCLEAR | LOW |
| **Hoda Et Al (26)** | HIGH | LOW | LOW | LOW |
| **Martens Et Al (19)** | LOW | UNCLEAR | LOW | UNCLEAR |
| **Introini Et Al (18)** | UNCLEAR | LOW | UNCLEAR | LOW |
| **Le Portz Et Al (17)** | HIGH | LOW | LOW | LOW |
| **Dikranian Et Al (16)** | LOW | HIGH | LOW | LOW |
| **Gregori Et Al (13)** | UNCLEAR | LOW | LOW | LOW |
| **Cestari Et Al (9)** | HIGH | LOW | LOW | LOW |
| **Van Uhm Et Al (38)** | LOW | LOW | LOW | UNCLEAR |
| **Cestari et al (39)** | UNCLEAR | LOW | LOW | UNCLEAR |
| **Stephen et al (40)** | HIGH | LOW | LOW | LOW |
| **Queissert et al (44)** | UNCLEAR | LOW | LOW | UNCLEAR |
| **Crivellaro et al (41)** | UNCLEAR | LOW | LOW | UNCLEAR |
