# Supplementary figures and images for: Comparison of Different Invasive Devices for the Treatment of Urinary Incontinence after Radical Prostatectomy
Source: Adv Urol. 2022 Jun 21;2022:8736249. doi: 10.1155/2022/8736249 (PMC9239822; doi:10.1155/2022/8736249)

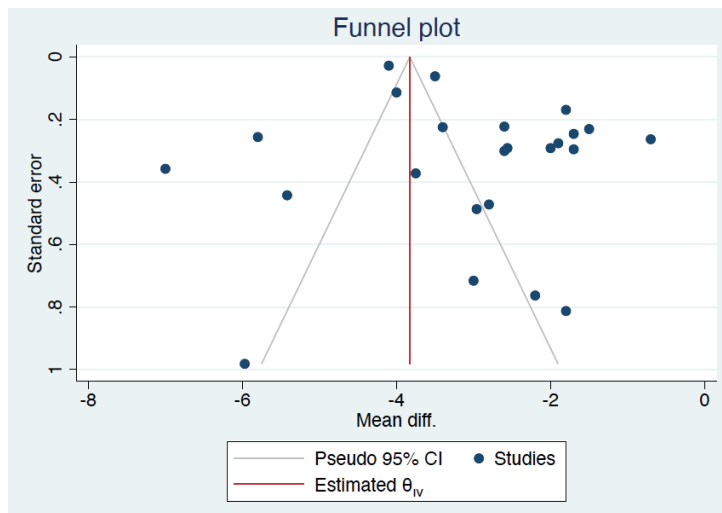

a)

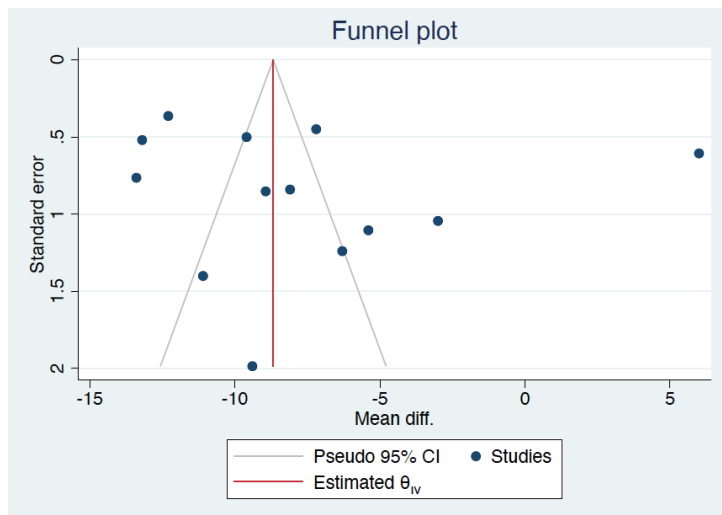

b)

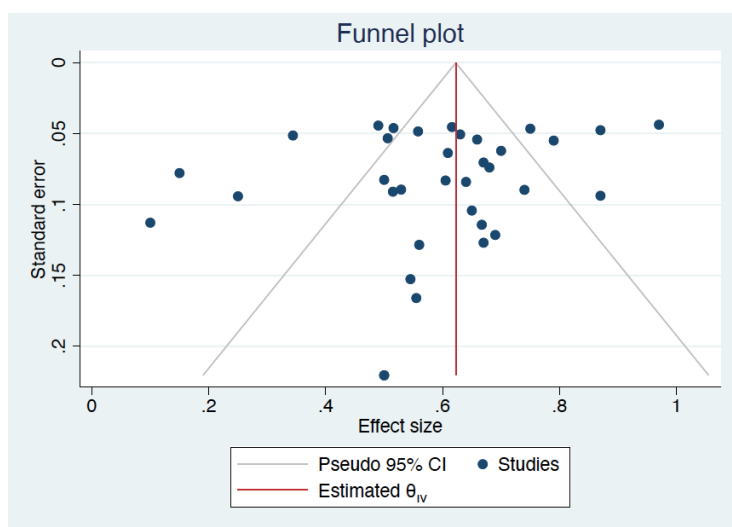

c)

Supplement: Supplementary Materials — Supplementary Figure 1. Flow chart for meta-analysis (PRISMA). Supplementary Figure 2. Deeks' funnel plots for standardized mean difference (SMD) of number of pad/day (a), ICIQ-SF score (b), and for continence event rate recovery (c) at follow-up. Supplementary Figure 3. Meta-regression plots in relation to standardized mean difference (SMD) for the number of pad (a), ICIQ-SF score (b), and for continence event rate (c) recovery at follow-up. Supplementary Table 1. Risk of Bias for all studies included in the meta-analysis. PRISMA checklist: checklist reporting location in the manuscript of the different items related to PRISMA analysis. [file 8736249.f1.zip › 8736249.f1/supplementary figure 2.pdf]

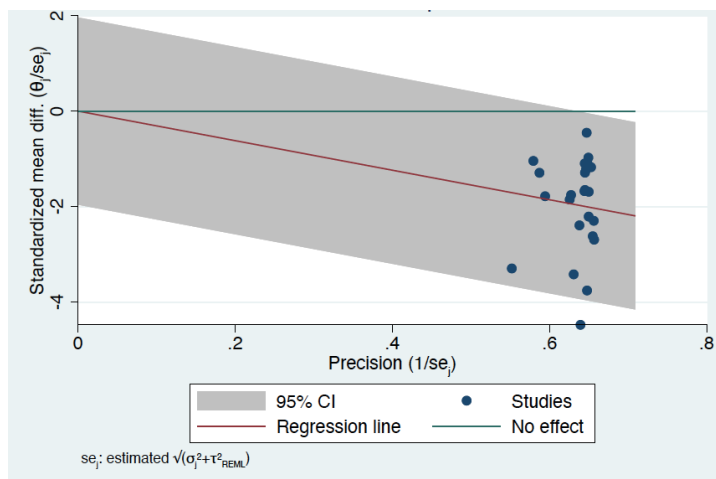

a)

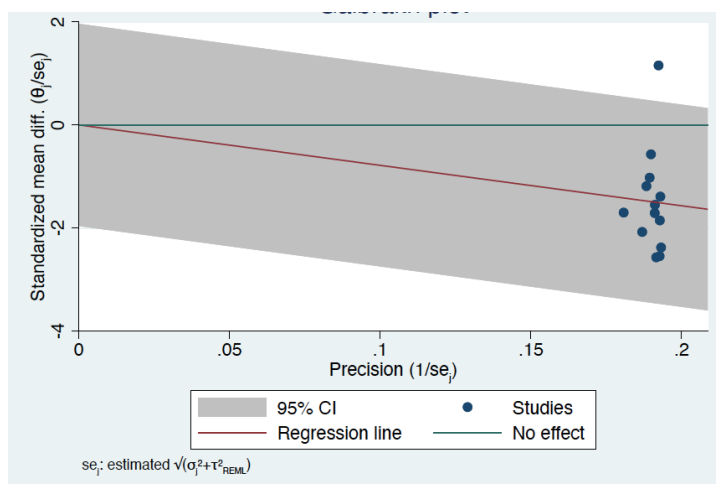

b)

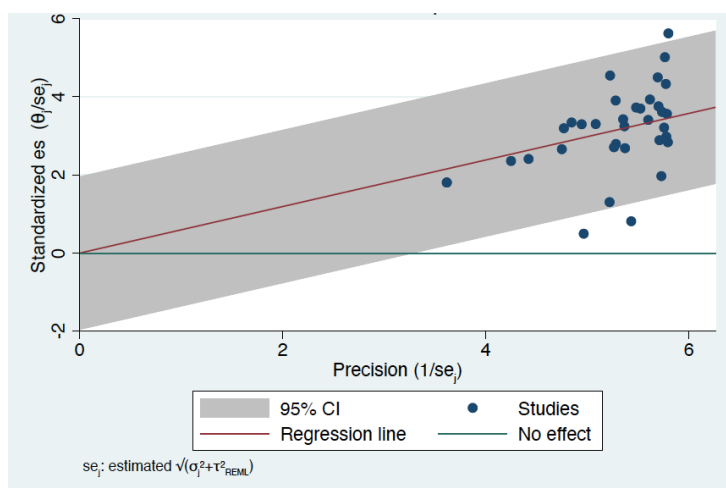

c)

Supplement: Supplementary Materials — Supplementary Figure 1. Flow chart for meta-analysis (PRISMA). Supplementary Figure 2. Deeks' funnel plots for standardized mean difference (SMD) of number of pad/day (a), ICIQ-SF score (b), and for continence event rate recovery (c) at follow-up. Supplementary Figure 3. Meta-regression plots in relation to standardized mean difference (SMD) for the number of pad (a), ICIQ-SF score (b), and for continence event rate (c) recovery at follow-up. Supplementary Table 1. Risk of Bias for all studies included in the meta-analysis. PRISMA checklist: checklist reporting location in the manuscript of the different items related to PRISMA analysis. [file 8736249.f1.zip › 8736249.f1/supplementary figure 3.pdf]
