# Supplementary material for: Comparison of Different Invasive Devices for the Treatment of Urinary Incontinence after Radical Prostatectomy
Source: Adv Urol. 2022 Jun 21;2022:8736249. doi: 10.1155/2022/8736249 (PMC9239822; doi:10.1155/2022/8736249)
Supplement: Supplementary Materials — Supplementary Figure 1. Flow chart for meta-analysis (PRISMA). Supplementary Figure 2. Deeks' funnel plots for standardized mean difference (SMD) of number of pad/day (a), ICIQ-SF score (b), and for continence event rate recovery (c) at follow-up. Supplementary Figure 3. Meta-regression plots in relation to standardized mean difference (SMD) for the number of pad (a), ICIQ-SF score (b), and for continence event rate (c) recovery at follow-up. Supplementary Table 1. Risk of Bias for all studies included in the meta-analysis. PRISMA checklist: checklist reporting location in the manuscript of the different items related to PRISMA analysis. [file 8736249.f1.zip › 8736249.f1/PRISMA_2020_checklist.doc]

| **Section and Topic** | **Item #** | **Checklist item** | **Location where item is reported** |
| --- | --- | --- | --- |
| **TITLE** | | |  |
| Title | 1 | Identify the report as a systematic review. | Yes on title page |
| **ABSTRACT** | | |  |
| Abstract | 2 | See the PRISMA 2020 for Abstracts checklist. | X yes on abstract form |
| **INTRODUCTION** | | |  |
| Rationale | 3 | Describe the rationale for the review in the context of existing knowledge. | Line56-72 |
| Objectives | 4 | Provide an explicit statement of the objective(s) or question(s) the review addresses. | Line 79-86 |
| **METHODS** | | |  |
| Eligibility criteria | 5 | Specify the inclusion and exclusion criteria for the review and how studies were grouped for the syntheses. | Line 90-93 and 101-106 |
| Information sources | 6 | Specify all databases, registers, websites, organisations, reference lists and other sources searched or consulted to identify studies. Specify the date when each source was last searched or consulted. | Line 89-90 |
| Search strategy | 7 | Present the full search strategies for all databases, registers and websites, including any filters and limits used. | Line 89-100 |
| Selection process | 8 | Specify the methods used to decide whether a study met the inclusion criteria of the review, including how many reviewers screened each record and each report retrieved, whether they worked independently, and if applicable, details of automation tools used in the process. | Line 89-100 |
| Data collection process | 9 | Specify the methods used to collect data from reports, including how many reviewers collected data from each report, whether they worked independently, any processes for obtaining or confirming data from study investigators, and if applicable, details of automation tools used in the process. | Line 89-100 |
| Data items | 10a | List and define all outcomes for which data were sought. Specify whether all results that were compatible with each outcome domain in each study were sought (e.g. for all measures, time points, analyses), and if not, the methods used to decide which results to collect. | Line 80-109 |
|  | 10b | List and define all other variables for which data were sought (e.g. participant and intervention characteristics, funding sources). Describe any assumptions made about any missing or unclear information. | Line 80-109 |
| Study risk of bias assessment | 11 | Specify the methods used to assess risk of bias in the included studies, including details of the tool(s) used, how many reviewers assessed each study and whether they worked independently, and if applicable, details of automation tools used in the process. | Line 114-121 and suppl table 1 |
| Effect measures | 12 | Specify for each outcome the effect measure(s) (e.g. risk ratio, mean difference) used in the synthesis or presentation of results. | Line 114-126 |
| Synthesis methods | 13a | Describe the processes used to decide which studies were eligible for each synthesis (e.g. tabulating the study intervention characteristics and comparing against the planned groups for each synthesis (item #5)). | Line 88-109 |
|  | 13b | Describe any methods required to prepare the data for presentation or synthesis, such as handling of missing summary statistics, or data conversions. | Line 88-109 |
|  | 13c | Describe any methods used to tabulate or visually display results of individual studies and syntheses. | Line 88-109 |
|  | 13d | Describe any methods used to synthesize results and provide a rationale for the choice(s). If meta-analysis was performed, describe the model(s), method(s) to identify the presence and extent of statistical heterogeneity, and software package(s) used. | Line 88-109 |
|  | 13e | Describe any methods used to explore possible causes of heterogeneity among study results (e.g. subgroup analysis, meta-regression). | Line 88-109 |
|  | 13f | Describe any sensitivity analyses conducted to assess robustness of the synthesized results. | Line 88-109 |
| Reporting bias assessment | 14 | Describe any methods used to assess risk of bias due to missing results in a synthesis (arising from reporting biases). | Line 114-116 and suppl table 1 |
| Certainty assessment | 15 | Describe any methods used to assess certainty (or confidence) in the body of evidence for an outcome. | Line 114-116 and suppl table 1 |
| **RESULTS** | | |  |
| Study selection | 16a | Describe the results of the search and selection process, from the number of records identified in the search to the number of studies included in the review, ideally using a flow diagram. | Line 128-152 |
|  | 16b | Cite studies that might appear to meet the inclusion criteria, but which were excluded, and explain why they were excluded. | Line 128-152 |
| Study characteristics | 17 | Cite each included study and present its characteristics. | Line 128-152 |
| Risk of bias in studies | 18 | Present assessments of risk of bias for each included study. | Line 128-152 |
| Results of individual studies | 19 | For all outcomes, present, for each study: (a) summary statistics for each group (where appropriate) and (b) an effect estimate and its precision (e.g. confidence/credible interval), ideally using structured tables or plots. | Line 154-186 |
| Results of syntheses | 20a | For each synthesis, briefly summarise the characteristics and risk of bias among contributing studies. | Line 154-186 suppl table 1 |
|  | 20b | Present results of all statistical syntheses conducted. If meta-analysis was done, present for each the summary estimate and its precision (e.g. confidence/credible interval) and measures of statistical heterogeneity. If comparing groups, describe the direction of the effect. | Line 190-227 table 1 and figure 1-2 |
|  | 20c | Present results of all investigations of possible causes of heterogeneity among study results. | Line 190-227, table 1 and figure 1-2 |
|  | 20d | Present results of all sensitivity analyses conducted to assess the robustness of the synthesized results. | Line 190-227 figure 1-2 |
| Reporting biases | 21 | Present assessments of risk of bias due to missing results (arising from reporting biases) for each synthesis assessed. | Line 190-227 figure 1-2 |
| Certainty of evidence | 22 | Present assessments of certainty (or confidence) in the body of evidence for each outcome assessed. | Line 190-227 |
| **DISCUSSION** | | |  |
| Discussion | 23a | Provide a general interpretation of the results in the context of other evidence. | Line 230-290 |
|  | 23b | Discuss any limitations of the evidence included in the review. | Line 240-290 |
|  | 23c | Discuss any limitations of the review processes used. | Line 240-290 |
|  | 23d | Discuss implications of the results for practice, policy, and future research. | Line 290-303 |
| **OTHER INFORMATION** | | |  |
| Registration and protocol | 24a | Provide registration information for the review, including register name and registration number, or state that the review was not registered. | Title page |
|  | 24b | Indicate where the review protocol can be accessed, or state that a protocol was not prepared. | Title page |
|  | 24c | Describe and explain any amendments to information provided at registration or in the protocol. | Title page |
| Support | 25 | Describe sources of financial or non-financial support for the review, and the role of the funders or sponsors in the review. | Title page |
| Competing interests | 26 | Declare any competing interests of review authors. | Title page |
| Availability of data, code and other materials | 27 | Report which of the following are publicly available and where they can be found: template data collection forms; data extracted from included studies; data used for all analyses; analytic code; any other materials used in the review. | Title page |

*From:*  Page MJ, McKenzie JE, Bossuyt PM, Boutron I, Hoffmann TC, Mulrow CD, et al. The PRISMA 2020 statement: an updated guideline for reporting systematic reviews. BMJ 2021;372:n71. doi: 10.1136/bmj.n71

For more information, visit: <http://www.prisma-statement.org/>
